# A Long-Term Comparison between the AethLabs MA350 and Aerosol Magee Scientific AE33 Black Carbon Monitors in the Greater Salt Lake City Metropolitan Area

**DOI:** 10.3390/s24030965

**Published:** 2024-02-01

**Authors:** Daniel L. Mendoza, L. Drew Hill, Jeffrey Blair, Erik T. Crosman

**Affiliations:** 1 Department of Atmospheric Sciences, University of Utah, 135 S 1460 E, Room 819, Salt Lake City, UT 84112, USA; 2 Pulmonary Division, School of Medicine, University of Utah, 26 N 1900 E, Salt Lake City, UT 84132, USA; 3 Department of City & Metropolitan Planning, University of Utah, 375 S 1530 E, Suite 220, Salt Lake City, UT 84112, USA; 4 AethLabs, 3085 21st Street, San Francisco, CA 94110, USA; drew.hill@aethlabs.com (L.D.H.); jeff.blair@aethlabs.com (J.B.); 5 Department of Life, Earth and Environmental Sciences, West Texas A&M University, Natural Sciences Building 324, Canyon, TX 79016, USA; etcrosman@wtamu.edu

**Keywords:** black carbon, aethalometer, AethLabs MA350, Aerosol Magee Scientific AE33, biomass, fossil fuel, blue wavelength, IR wavelength, sensor comparison, public health

## Abstract

Black carbon (BC) or soot contains ultrafine combustion particles that are associated with a wide range of health impacts, leading to respiratory and cardiovascular diseases. Both long-term and short-term health impacts of BC have been documented, with even low-level exposures to BC resulting in negative health outcomes for vulnerable groups. Two aethalometers—AethLabs MA350 and Aerosol Magee Scientific AE33—were co-located at a Utah Division of Air Quality site in Bountiful, Utah for just under a year. The aethalometer comparison showed a close relationship between instruments for IR BC, Blue BC, and fossil fuel source-specific BC estimates. The biomass source-specific BC estimates were markedly different between instruments at the minute and hour scale but became more similar and perhaps less-affected by high-leverage outliers at the daily time scale. The greater inter-device difference for biomass BC may have been confounded by very low biomass-specific BC concentrations during the study period. These findings at a mountainous, high-elevation, Greater Salt Lake City Area site support previous study results and broaden the body of evidence validating the performance of the MA350.

## 1. Introduction

### 1.1. Background on Black Carbon

Black carbon (BC) or soot contains ultrafine combustion particles that are associated with a wide range of health impacts leading to respiratory and cardiovascular diseases [1,2]. Both long-term and short-term health impacts of BC have been documented, with even low-level exposures to BC resulting in negative health outcomes for vulnerable groups [3]. As summarized by Watson et al. [4], various instrumentation techniques exist in the literature for estimating BC, including both thermal and optical analysis methods. Optical BC measurement techniques using aerosol light absorption have been in use for over four decades [5]. The most commonly used instrument for optical BC measurement is the aethalometer, which actively collects aerosols on a filter and measures the resulting attenuation of transmitted light [5]. In this study, we conduct a comparison of the AethLabs MA350 (“MA350”) [6] and Aerosol Magee Scientific AE33 (“AE33”) [7] BC aethalometer instruments. The AE33 [7] is among the most-widely used instruments for real-time monitoring and speciation of aerosol BC. The MA350 [6] is a smaller “micro” aethalometer that is designed for installation at remote and inaccessible stationary sites as well as for mobile use cases. To these ends, it is small, lightweight, and capable of extended battery-powered operation. As a result, the MA350’s sample spot size is smaller, its flow rate is lower (up to 0.170 L min^−1^ vs. up to 5 L min^−1^ for the AE33), and face velocity at the sample spot is much reduced relative to the AE33. With this in mind, and because the AE33 is a rack-mount monitor costing substantially more than the MA350 and with a lineage rooted in a longer history of widespread use, our primary goal in this study is to compare the performance of the MA350 against the AE33 at various time scales (primarily minute, hour, and day) to characterize its use for health and policy studies in the Greater Salt Lake City Metropolitan Area.

### 1.2. Source Apportionment and “Aethalometer Model”

Several aethalometer products offer onboard source apportionments. Typically, this method is based on the “Aethalometer Model” of source apportionment estimation, which, to our knowledge, was introduced by Sandradewi et al. [8] and is the same general method currently implemented by the AE33 and MA350 [9].

The Aethalometer Model, and the theory and experimentation that support it are described in depth by Sandradewi et al. [8], Martinsson et al. [10], Zotter et al. [11], Helin et al. [12], and Sandradewi et al. [13] and numerous studies have employed it, e.g., Sandradewi et al. [5] and Favez et al. [14]. The Sandradewi et al. [13] article, for example, has been cited over 430 times to date. Briefly, the Aethalometer Model leverages simultaneous optical measurements in the UV or blue (“Blue”) and near-IR (“IR”) ranges to quantify carbonaceous aerosol concentrations from wood combustion sources and, separately, fossil fuel combustion sources. To set a baseline for expected comparative source apportionment performance, we compare the results of output derived from the Aethalometer Model for the MA350 and AE33.

## 2. Materials and Methods

### 2.1. Location and Study Period

The aethalometers were co-located at the Utah Division of Air Quality (UDAQ) Bountiful station located at: 40.902945° N, 111.884505° W, 1309 masl [15]. The AE33 (Aerosol Magee Scientific, Berkeley, CA, USA) unit [7] is maintained by UDAQ and has been in operation for multiple years at this site. The MA350 (AethLabs, San Francisco, CA, USA) unit [6] was installed on 30 August 2021 and data were collected until 8 August 2022. The UDAQ trailer in which the AE33 and MA350 were housed is temperature controlled.

### 2.2. Instrument Operation and Parameters

The MA350 and AE33 are both filter-based light attenuation monitors, and both implement variations of the DualSpot^®^ approach to compensate measurements for filter loading effects—or the reduction of the rate of light attenuation per unit of deposited BC mass at high levels of filter loading (or high values in the “ATN” related data fields), which is attributed to a shadowing effect caused by the accumulation of particles on and in the filter [16]. Generally, the DualSpot^®^ approach, which is discussed in more detail in Drinovec et al. [17] compares measurements from two sample spots on the filter that are made to have different loading rates through differential flow rates (greater flow through Spot1). A compensation parameter, “k”, is then applied to the Spot1 data to produce a compensated BC output. The equations for k used in the MA350 and AE33 differ marginally. To the best of our knowledge, the procedure and equations for calculating k and compensated BC currently used in the AE33 are described in Drinovec et al. [17]. In the MA350, k and compensated BC are calculated using principles discussed in the literature [16,17,18] and using Equations (1) and (2), respectively, previously published in the supplemental materials of Chakraborty et al. [19].

(1) $$k_{MA350}=\frac{{BC}_{Spot2}-{BC}_{Spot1}}{\left({BC}_{Spot2}\cdot {ATN}_{Spot1}\right)-\left({BC}_{Spot1}\cdot {ATN}_{Spot2}\right)}$$

(2) $${BCc}_{MA350}=\frac{{BC}_{Spot1}}{1-\left(k\cdot {ATN}_{Spot1}\right)}$$

**Figure 1:**
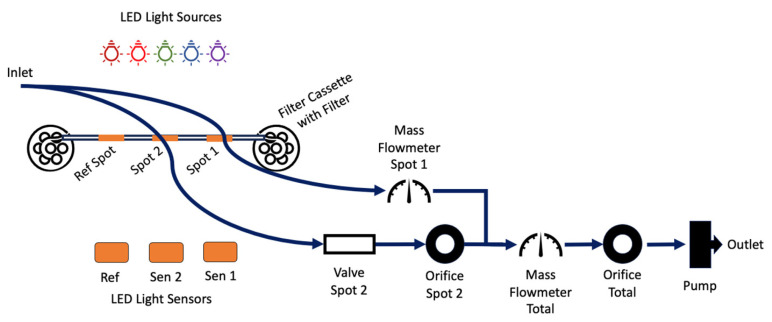
Diagrammatic representation of the path that sample air takes through the MA350, showing high-level components.

A flow diagram representing the key measurement components of the MA350 is shown in Figure 1. A similar flow diagram for the AE33 has been published in Drinovec et al., 2015 [17].

The AE33 was operated at 60 s timebase and 5 L min^−1^ flow rate with DualSpot^®^ compensation. The MA350 was operated at a 60 s timebase and 0.150 L min^−1^ total flow rate in DualSpot^®^ mode and used a pre-release firmware, v1.11, with an early version of the source apportionment model implemented, which has since been revised and released via firmware v1.12 and later). Instrument features are summarized in Table 1. Unless otherwise stated, the BC data discussed in this study have been compensated for filter loading effects. AE33 data were collected using the United States Environmental Protection Agency (USEPA) flow reporting standard of 101,325 Pa and 25 °C.

### 2.3. Data Treatment

**Figure 2:**
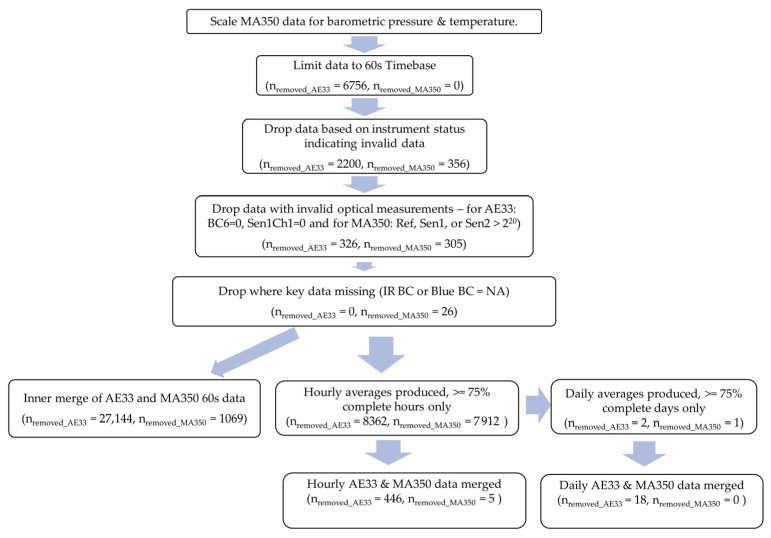
Flow diagram of the data cleaning approach with number of datapoints removed from each device dataset at each step.

Data for the study period was collected, cleaned, and processed using the procedures described below. Cleaning and analysis were performed using Python 3.11.3 (Fredricksburg, VA, USA) and R version 4.3.1 (The R Foundation, Indianapolis, IN, USA) [22]. The R code used is available to anyone interested via request from the corresponding author. A summary of the data cleaning procedure is shown in Figure 2.

#### 2.3.1. AE33 Data Cleaning

The dataset was limited to a 60 s timebase by removing 1 s timebase data (n_removed_ = 6756). A check for duplicate time stamps revealed none. Datapoints with concerning statuses were dropped (n_removed_ = 2200). A list of possible instrument status states identified in this study as potentially concerning as well as whether they were observed in the dataset is shown in Table 2. Data that had BC6 or Sen1Ch1 field values equal to 0 were also removed (n_removed_ = 326). Lastly, records with potentially invalid values of k, the loading compensation correction values (k < −0.005 or k > 0.015), would have been removed [23], but none were found.

#### 2.3.2. MA350 Data Cleaning & Processing

The first step was to adjust flow values for “Sample temp (°C)” and “Internal pressure (Pa)” to account for site specific conditions, as the MA350’s internal flow calibration table was not recalibrated onsite prior to deployment. While AethLabs’ firmware (v1.12 and later) now reports additional variables with the sample temperature and instrument pressure from the most recent flow calibration, the firmware used in this study did not, and so this adjustment procedure was performed using typical values for temperature (25 °C–~20 °C for room temperature and ~5 °C for heat produced by the instrument during sampling) and pressure (101,325 Pa) for the location of the most recent prior calibration: San Francisco, CA, which also happens to be the USEPA flow reporting standard conditions. The mean barometric pressure for the study period was measured to be 86,467 Pa. The adjustment equation is derived from the Ideal Gas Law and was applied for every data point. Specifically, a ratio of the original “Flow total (mL/min)” measurement to its adjusted value was created for each datapoint and used to scale corresponding flow values and values derived therefrom (e.g., BC mass concentration).

The dataset did not have any 1 s timebase data or duplicate timestamps. As was performed for the AE33 processing, 356 data points with concerning instrument status list values were dropped (Table 3). In total, 305 data points with high MA350 optical values (greater than 2^20^) were removed because that may be an indication of possible invalid data. Source apportionment data were calculated for the remaining data.

#### 2.3.3. Hourly and Daily Averaging

After the above data cleaning steps, the 60 s timebase data comprising each hour were assessed for completeness, and any hours with <75% completeness (<45 datapoints per hourly average) were dropped. The resulting hourly data were used to produce daily averages after assessment for completeness—any days with <75% completeness (<18 datapoints per daily average) were dropped.

### 2.4. Source Apportionment

#### 2.4.1. Theoretical Basis for Source Apportionment

The Aethalometer Model compares concurrent measurements at specific short wavelengths (usually UV or Blue) and near-IR (“IR”) to estimate BC concentrations produced by wood combustion relative to BC concentrations produced by fossil fuel combustion sources. Wood smoke aerosols are rich in organic compounds which absorb light more strongly in shorter wavelengths (like UV and blue) than at the traditional wavelengths used to assess BC (IR). Traffic-related aerosols and soot, on the other hand, comprise significantly smaller fractions of organics and, due to their purer BC composition, absorb light across the UV-IR range at a strength directly proportional to wavelength^−1^, or 1/λ where λ represents wavelength [8].

This spectral dependence across the UV-IR range can be parameterized by the Ångström exponent (AAE), defined as 1 for aerosols with a consistent 1/λ spectral dependence in absorptivity (pure BC), >1 when light absorption is stronger at shorter wavelengths than expected under a 1/λ dependence, and <1 when light absorption is stronger at longer wavelengths than expected under a 1/λ dependence. In measurement areas where BC comes only from wood combustion and fossil fuel combustion, it is this spectral dependence of light absorption (AAE) that allows the aethalometer model to delineate the proportion of a sampled aerosol from wood burning sources relative to the proportion from fossil fuel combustion.

The equations that underly the Aethalometer Model are produced using Beer–Lambert’s Law and take as input several parameters that can be measured using a multi-wavelength aethalometer like the MA350. At a high level, aethalometers illuminate a filter spot with a specific wavelength of light (usually ~880 nm) to measure the change in optical attenuation as particles accumulate on the filter. The filter spot surface area is known, and the volume of air that passes through the filter spot per unit time is measured; these metrics are used to calculate the mass concentration of BC particles in a cubic meter of air [24]. Under low filter loading (low attenuation), this attenuation is proportional to the BC mass deposited onto the filter allowing for a BC mass concentration to be obtained [25]—after potential multiple scattering effects inherent in the specific filter medium being used have been accounted for with an empirically calculated constant (C_ref_) [16]. From this mass concentration, C_ref_, and an a priori knowledge of the instrument’s wavelength-specific mass absorption cross section (MAC or σ_abs_), a wavelength-specific aerosol absorption coefficient (b_abs_) can be obtained for the aerosol sample. Together, these concepts laid the groundwork for the Aethalometer Model.

#### 2.4.2. Mathematical Foundations for the MA350 Source Apportionment Feature

At the time of instrument deployment, AethLabs was in the process of developing a firmware-based source apportionment estimation feature for its microAeth^®^ MA Series of instruments. The specific algorithm used to implement this feature was in beta and has since been slightly revised. We have implemented a close approximation of the method used in the current firmware (v1.12 and later) using post-processing to mimic the output of the publicly released feature and facilitate discussion and realistic inter-device comparison of source apportionment output.

We applied the Aethalometer Model to MA350 measurements taken at 470 nm (“Blue”) and 880 nm (“IR”) [6,19]. The values of C_ref_, MAC_470nm_, and MAC_880nm_ for the microAeth MA Series have been calculated by AethLabs at 1.3 m^2^ g^−1^, 10.120 m^2^ g^−1^, and 19.070 m^2^ g^−1^, respectively [26]. Thus, total aerosol absorptions coefficients for the wavelengths 470 nm (b_abs,470nm_) and 880 nm (b_abs,880nm_) for an MA350 BC sample can be calculated using Equations (3) and (4) [16,24], where Blue BCc and IR BCc are the BC mass concentrations measured in the blue (470 nm) and IR (880 nm) wavelengths, respectively. Both the Blue BCc and IR BCc measurements are compensated for filter loading effects using a DualSpot^®^-based approach, described in Section 2.2.

(3) $$b_{\mathrm{abs},470\mathrm{nm}}=\mathrm{Blue}\_\mathrm{BCc}\cdot \frac{{\mathrm{MAC}}_{470\mathrm{nm}}}{C_{\mathrm{ref}}}$$

(4) $$b_{abs,880nm}=IR\_BCc\cdot \frac{{MAC}_{880\mathrm{nm}}}{C_{ref}}$$

The total AAE of an aerosol sample is a parameter that is highly dependent upon the aerosol’s composition and, thus, its source [8,11]. It is a pivotal part of the Aethalometer Model of source apportionment. AAE can be calculated using Equation (5).

(5) $$AAE=-\frac{\ln\left(\frac{b_{abs}(470nm)}{b_{abs}(880nm)}\right)}{\ln\left(\frac{470}{880}\right)}$$

The source-specific absorption coefficients for aerosols from wood combustion sources (b_abs,wb,880nm_) and fossil fuel combustion sources (b_abs,ff,880nm_) are calculated for 880 nm using Equations (6) [11] and (7) [5], respectively.

(6) $$b_{abs,ff,880nm}=\frac{b_{abs}\left(470nm\right)-b_{abs}(880nm)\cdot {\left(\frac{470}{880}\right)}^{{-AAE}_{wb}}}{{\left(\frac{470}{880}\right)}^{{-AAE}_{ff}}-{\left(\frac{470}{880}\right)}^{{-AAE}_{wb}}}$$

(7) $$b_{abs,wb,880nm}=b_{abs,470nm}-b_{abs,ff}$$

AAE_wb_ and AAE_ff_ values vary by the nature of the sources that produce an aerosol sample [27], as factors like fuel type, characteristics of the combustion event and technology, and atmospheric aging will affect the refractory properties of an aerosol. It is thus important to consider the specific local and regional combustion sources when selecting AAE_wb_ and AAE_ff_ values for analysis. Several studies have measured the AAE values of source-specific emissions for wavelengths similar to those used in our analysis (470 nm and 880 nm), however, to our knowledge, no accepted standard values exist for our sample area and so we must estimate these parameters using local and regional context.

The AAE of wood burning emissions (AAE_wb_) is commonly reported in a range that extends from about 1 to upwards of about 5 [10,11,14,27], while the AAE of emissions from fossil fuel sources (AAE_ff_) appears to be less variable and has been observed close to 1 [11]—as is expected for aerosols comprised of nearly pure BC. Residential heating and recreational fires [28] are the main local wood combustion sources. Regional sources and likely the majority of wood burning aerosols in our sample area stem from wildfire smoke [29]—likely transported from California and the Pacific Northwest given local wind patterns—especially with the recent advent of statewide wood stove and fireplace conversion assistance programs [30]. Some agricultural burning in Northern Utah may also impact our samples in summer months [28,31]. Local fossil fuel combustion sources are comprised of highway and residential traffic with contributions from local industrial facilities [15,32].

The proportion of measured BC mass concentration attributed to wood burning is calculated as a percentage (BB%) using Equation (8) [12].

(8) $$BB\%=\frac{b_{abs,wb,880nm}}{b_{abs,880nm}}\cdot 100$$

The BC mass concentrations from wood burning (BC_wb_) and fossil fuel sources (BC_ff_) are calculated using Equations (9) and (10), respectively [12].

(9) $${BC}_{wb}=\frac{BB\%\cdot IR\_BCc}{100}$$

(10) $${BC}_{ff}=\frac{\left(1-BB\%\right)\cdot IR\_BCc}{100}$$

A double exponentially weighted moving average (“DEMA”) is applied during calculation of source apportionment variables to reduce noise-induced artifacts using Equations (11) and (12) [31] while limiting lag, where EMA(X) is an exponentially weighted moving average of a sample X, EMA(X_t−1_) is an exponentially weighted moving average of the previously taken contiguous sample, DEMA(X) is a double exponentially weighted moving average of a sample X, and α is a smoothing parameter.

(11) $$EMA\left(X\right)=\left(1-\propto \right)\cdot EMA\left(X_{t-1}\right)+\propto \cdot X$$

(12) $$DEMA\left(X\right)=\left(2\cdot EMA\left(X\right)\right)-EMA(EMA\left(X\right))$$

**Figure 3:**
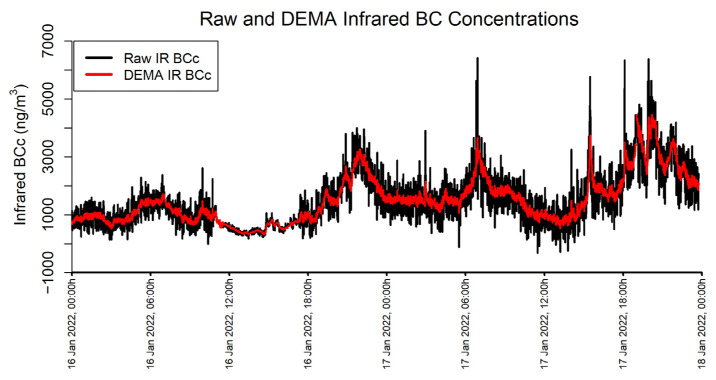
Raw and DEMA Infrared BC Concentrations.

An α of 0.125 is used on measurements with a timebase of 60 s to approximate a smoothing window of about 15 min, or 900 s, using the Equation 2/(N+1) where N is the desired smoothing period (in this case N = 900 s/60 s). An example of the MA350 BC data collected during this study is shown in Figure 3 in their raw state (“IR BCc”) and as DEMA-smoothed according to the process described above (“IR BCc DEMA”).

Noise-induced artifacts are further reduced by limiting BB% to logical values between 0 and 100 by coercing negative values to 0 and values above 100 to 100.

## 3. Results

**Figure 4:**
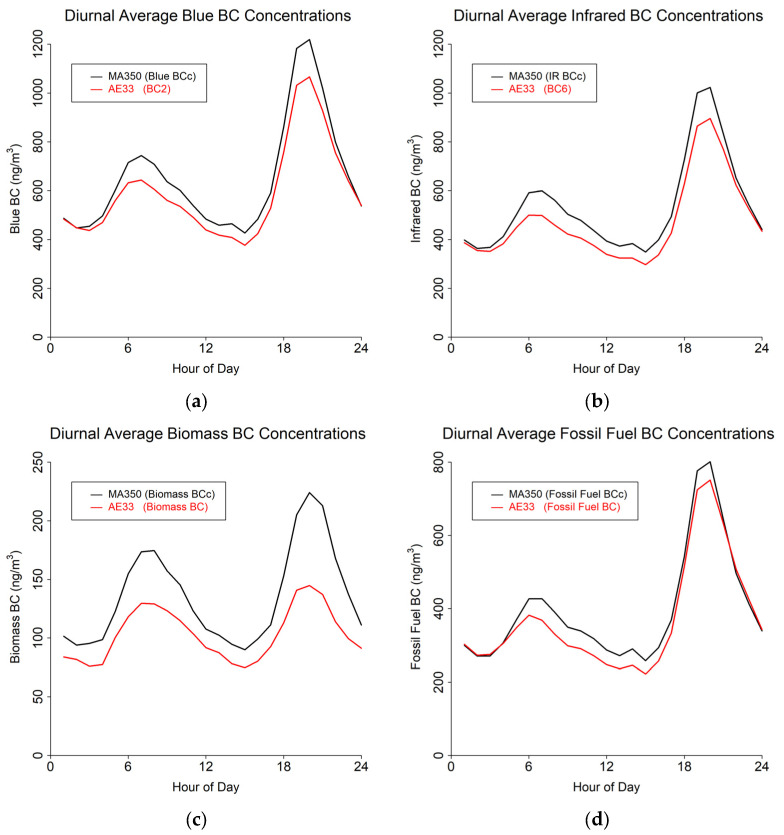
Diurnal cycles from 60 s timebase data for MA350 and AE33: (**a**) Blue wavelength, (**b**) Infrared wavelength, (**c**) Calculated biomass BC concentrations, and (**d**) Calculated fossil fuel BC concentrations.

**Figure 5:**
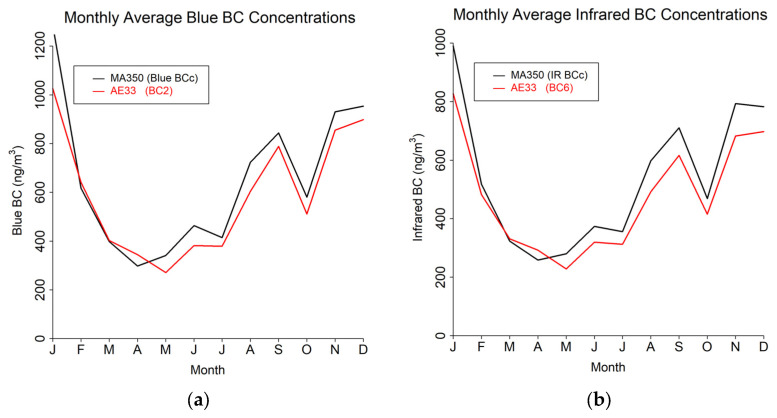

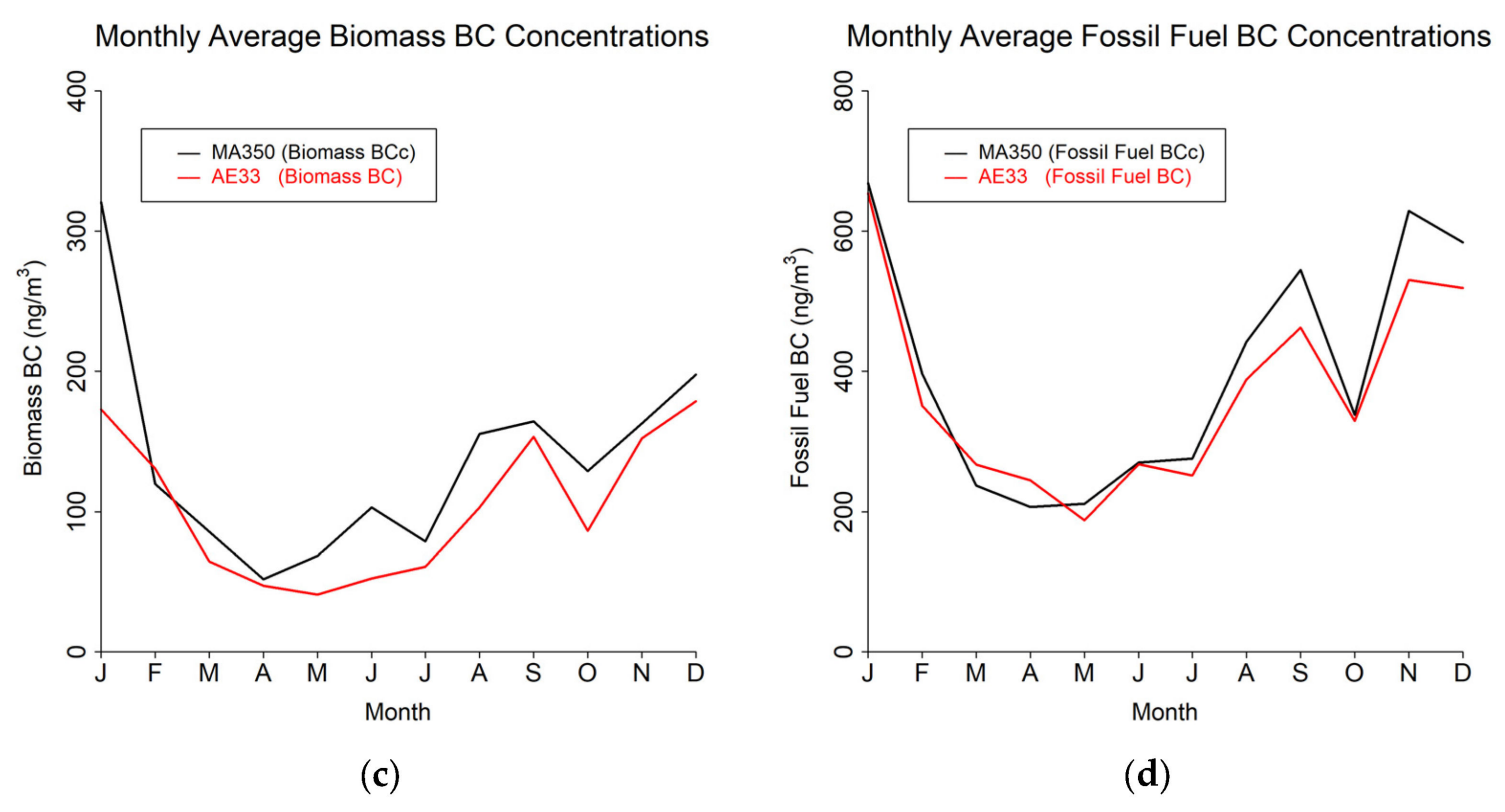
Monthly trends from 60 s timebase data for MA350 and AE33: (**a**) Blue wavelength, (**b**) Infrared wavelength, (**c**) Calculated biomass BC concentrations, and (**d**) Calculated fossil fuel BC concentrations.

**Figure 6:**
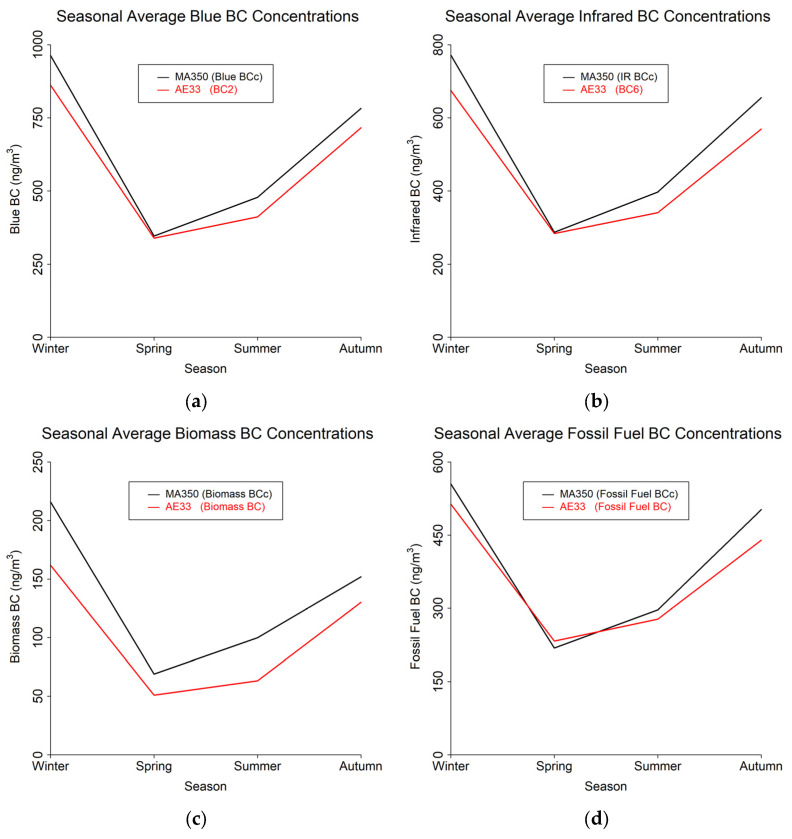
Seasonal trends from 60 s timebase data for MA350 and AE33: (**a**) Blue wavelength, (**b**) Infrared wavelength, (**c**) Calculated biomass BC concentrations, and (**d**) Calculated fossil fuel BC concentrations.

The study results are presented in terms of three distinct temporal averaging intervals: minute, hourly, and daily. Table 4 shows summary statistics for the entire study period using the 60 s timebase. Figure 4, Figure 5 and Figure 6 show mean diurnal, monthly, and seasonal trends, respectively, for Blue BC (Figure 4a, Figure 5a and Figure 6a), IR BC (Figure 4b, Figure 5b and Figure 6b), Biomass BC (Figure 4c, Figure 5c and Figure 6c), and Fossil fuel BC (Figure 4d, Figure 5d and Figure 6d) for the entire study period as created from 60 s timebase data. Seasons were defined as: Winter (December, January, February), Spring (March, April, May), Summer (June, July, August), and Autumn (September, October, November). Most BC measurements are reported in hourly or daily values for health studies. Because of the high temporal variability in BC measurements, post-processing methods to reduce noise are often developed.

### 3.1. Minute Resolved Findings

**Figure 7:**
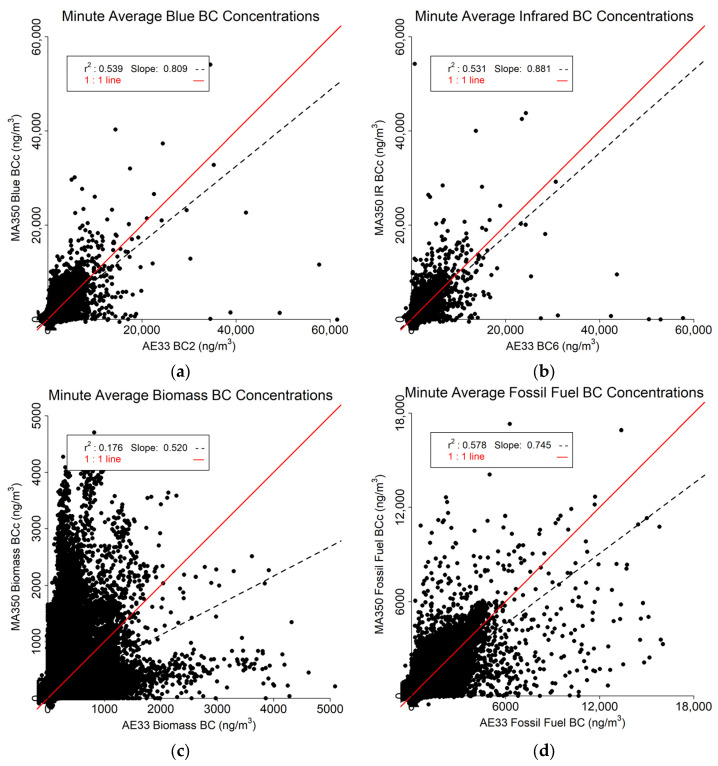
Minute resolved comparison between MA350 and AE33: (**a**) Blue wavelength, (**b**) Infrared wavelength, (**c**) Calculated biomass BC concentrations, and (**d**) Calculated fossil fuel BC concentrations.

The results from the minute-resolved data are shown in Figure 7. It was expected that the minute data would be relatively noisy, However, both the Blue (Figure 7a) and IR (Figure 7b) data correspond relatively well between the MA350 and AE33 (r^2^ > 0.530). The Biomass BC r^2^ value (Figure 7c) is comparatively low due to many outliers, However, the Fossil Fuel BC r^2^ value (Figure 7d) is moderate (0.578). For all four variables, the regression slope is less than one indicating that the MA350 underestimates the AE33 readings.

### 3.2. Hourly Averaged Findings

**Figure 8:**
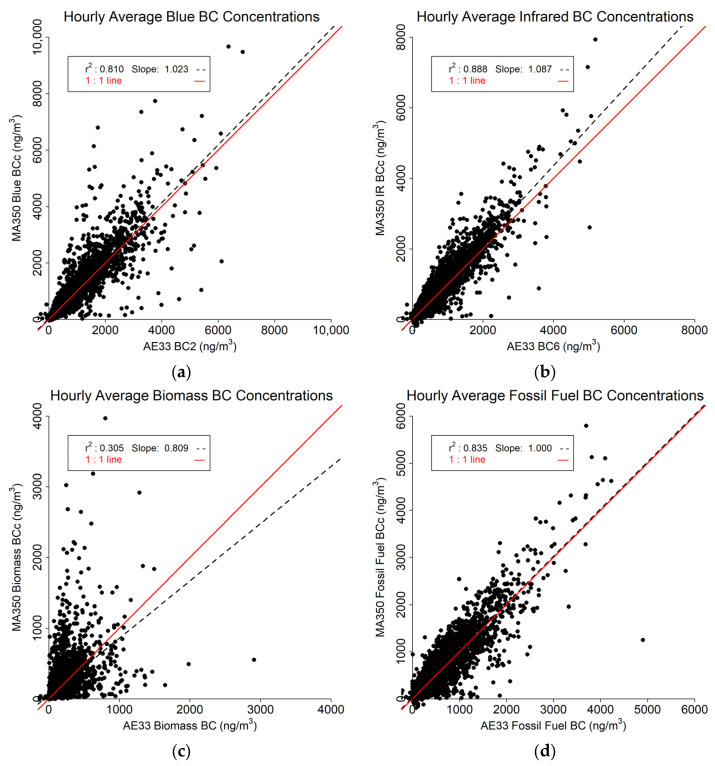
Hourly averaged data comparison between MA350 and AE33: (**a**) Blue wavelength, (**b**) Infrared wavelength, (**c**) Calculated biomass BC concentrations, and (**d**) Calculated fossil fuel BC concentrations.

The hourly averaged results (Figure 8) are less impacted by the effects of outliers. The Blue (Figure 8a) and IR (Figure 8b) BC readings between the two instruments compare favorably with r^2^ of 0.810 and 0.888, respectively. Regression slopes indicate the MA350 slightly overestimates the AE33 for Blue BC (by about 2%, slope = 1.023), and a bit more for Blue IR BC (about 9%, slope = 1.087). The Biomass BC (Figure 8c) comparison shows substantially lower r^2^ value (0.305), However, the Fossil Fuel BC estimates (Figure 8d) are highly similar (r^2^ = 0.835 with a slope of 1, or no typical bias).

### 3.3. Daily Averaged Findings

**Figure 9:**
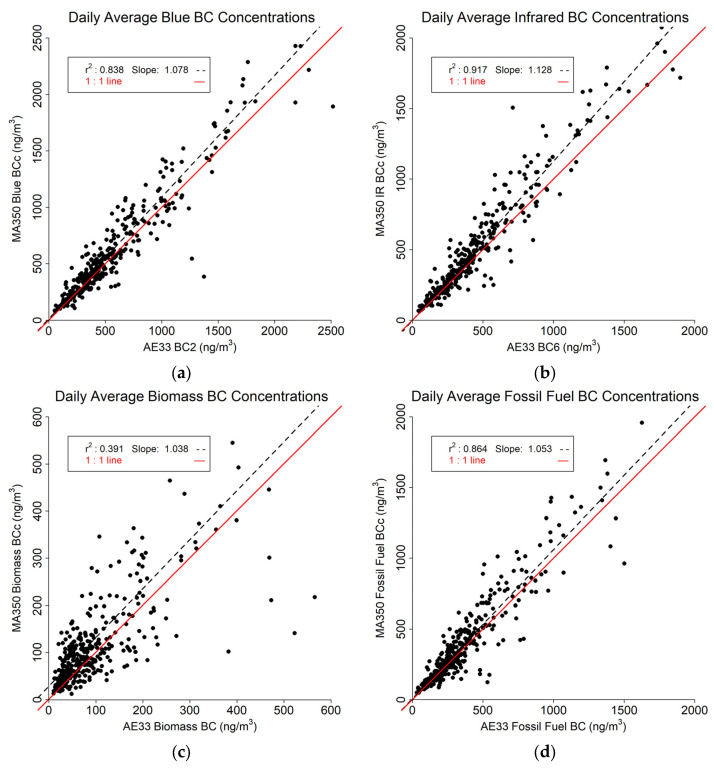
Daily averaged data comparison between MA350 and AE33: (**a**) Blue wavelength, (**b**) Infrared wavelength, (**c**) Calculated biomass BC concentrations, and (**d**) Calculated fossil fuel BC concentrations.

The daily average results are shown in Figure 9. As expected, the daily averaged values show much closer correlations across the two instruments. The Blue BC (Figure 9a) and IR BC (Figure 9b) readings both show a slope greater than one with no difference from hourly averaged values and strong correlation between the two instruments with r^2^ of 0.838 and 0.917 for Blue BC and IR BC, respectively. The Biomass estimates (Figure 9c) also show a positive slope, and as for all timebases, the lowest r^2^ (0.391). The Fossil Fuel comparison (Figure 9d) shows an r^2^ of 0.864 and a slope of 1.053.

## 4. Discussion

### 4.1. Implications

The aethalometer comparison showed a high level of agreement between the two instruments’ IR BC, Blue BC, and fossil fuel BC estimates. The biomass BC estimates were markedly different between devices at the minute and hour scale but became more similar at the daily, monthly, and seasonal timescales. The increasing trend in correlation as averaging time is increased suggests the 60 s data may be affected by a few high-leverage outliers. Such outliers and perhaps even the inter-device difference in BC biomass magnitude (i.e., slope coefficient) at the more-frequent levels of aggregation may be due to very low concentrations during the study period—especially for biomass BC (mean AE33-reported biomass BC = 103 ng m^−3^). The true biomass BC values may be near the limit of detection for the Aethalometer Model at the 60 s timebase of one or both instruments. This would result in the blue-IR differential being more heavily influenced by noise than it otherwise might be. The biomass calculation is sensitive to noise, and thus the fraction of biomass burning may be less stable or non-discernable when measuring low BC concentrations, low biomass-specific BC concentrations, or low fossil fuel-specific BC concentrations. Improved correlation may be observed at higher timebases, such as the 300 s timebase offered in both instruments’ stock settings. This may be especially true for the MA350, which has a sample spot face velocity that is often around 5–6 times lower than that of the AE33 due to the AE33’s much higher air flow rate.

Another interesting feature of the data is that at the minute scale, the MA350-AE33 regression slope was less than one, indicating an underestimation of AE33 by MA350, but increased progressively under the hourly and daily averaging, crossing one to produce a bias in the opposite direction. Diurnal patterns—which represent much longer averaging periods—displayed a consistent overestimation of the AE33 output by the MA350. The two devices track each other well throughout the day, outlining the same major temporal trends during the period of study.

Our results are in line with previous analyses comparing the AethLabs microAeth family of instruments with the AE33. For example, Kuula et al. [33] collocated an MA350 and AE33 at the same flow setpoints and timebases as our study for correlation at Blue and IR wavelengths of r^2^ = 0.98 and r^2^ = 0.97, respectively, and regression slopes of 0.91 and 0.85, respectively. Biomass BC and fossil fuel BC comparisons produced an r^2^ of 0.92 and 0.90, respectively, and slopes of 1 and 0.81, respectively. These results indicate better agreement between the two devices than do ours, which we hypothesize may be due to the difference in how our study and Kuula et al. [33] treat extreme BB% values. Their analysis removed about 14% of their data due to BB% extreme values, whereas our implementation retained such data but allocated them to 0% (negative values) or 100% (values > 100%). In our data, this rule affected about 26% of the final cleaned MA350 60 s datapoints. Blanco–Donado et al. [34] observed a 9% difference between a collocated MA200 (which was the lower reporter) and AE33 during an 80 h ambient measurement cycle in Colombia while using the same flow set points and timebase as our study as well as aerosol inlet driers.

It should be noted that AE33 units may differ from each other at a magnitude similar to the MA350-AE33 differences observed in our analysis [35]. A recent intra-device comparison of 23 AE33 units showed that it is not uncommon for IR BC measurements from well-maintained AE33 units to differ by 1–17% from one another while measuring ambient air. Our inter-device comparison for IR BC is within this range.

### 4.2. Limitations

The Aethalometer Model of source apportionment requires that the BC sources in one’s measurement region be comprised fully by wood combustion and fossil fuel combustion. This assumption is reasonable in our sample area because the largest sources are diesel vehicle emissions from the nearby Interstate 15 highway and wildfire emissions. Therefore, we believe our source apportionment estimates are valid. Biogenic sources may be a third considerable source of carbonaceous aerosols, however, they are likely non light-absorbing and thus unlikely to contribute substantially to bias in our analysis [10].

Our analysis may have benefitted from an onsite flow recalibration of the MA350 prior to sampling, and future work should include onsite flow calibrations as pressure and temperature values change considerably. A sensitivity analysis suggested negligible effects from assuming a standard pressure and temperature for the instrument’s true underlying San Francisco calibration conditions. For example, a ±5% change in pressure assumption alters the % mean inter-device difference estimates for IR BC and Biomass BC by about ±4–5 points (e.g., the reported IR BC inter-device difference is 11%, with a range of 7–16% based on a ±5% uncertainty in flow calibration pressure), which would not considerably change our primary findings. The effects are similar for a ±5 °C change in assumed sample temperature at calibration, with a ±1–2 point effect on estimated inter-device mean % difference.

### 4.3. Health and Policy Applications

Although high correlation at the minute scale would be ideal, realistically, high BC measurements are highly variable on minute time scales, and the overarching events, such as rush hour traffic, diurnal residential heating patterns, and wildfires span multiple hours, or, for some events, days [36]. Therefore, the overall and source-specific BC measurements during these episodes can be captured using higher timebases and hourly or daily averaging. The close correlation observed at the longer timescales should provide sufficient detail to capture the temporal patterns.

As the health hazards associated with BC exposure are being studied more and understood more fully, it is critical to develop a more extensive observation network. Since the MA350 is portable, robust, and significantly less expensive than monitors traditionally used at regulatory stations, these instruments may enhance monitoring efforts with relatively high accuracy. The increased fossil fuel signal during the winter season is consistent with atmospheric inversion periods that take place in Utah [37] trapping pollutants within the troposphere. The autumn biomass may be attributable to wildfire emissions [38]. Wildfires are increasing in frequency and magnitude and result in substantial economic [39] and health repercussions [40]. A network of BC sensors, coupled with atmospheric dispersion models [41], would provide invaluable information to public health departments to inform and help protect vulnerable community members and provide more insights into seasonal variations and long-term trends.

## 5. Conclusions

We collocated an AethLabs MA350 with an Aerosol Magee Scientific AE33 for just under a year and compared IR, Blue, and derived Biomass and Fossil Fuel black carbon concentrations. While the AE33 is among the most-widely used instruments for real-time monitoring and speciation of aerosol BC, the small and highly portable MA350 designed for installation at remote and inaccessible stationary sites and for mobile use cases has been underutilized in the field. The portable, robust, and significantly less expensive MA350 (compared to regulatory monitors), shows promise in being used to enhance black carbon monitoring efforts with relatively high accuracy.

This study demonstrates a close relationship between the MA350 and AE33 instruments for IR BC, Blue BC, and fossil fuel source-specific BC estimates. The 1 min timebase showed promising results. The hourly averaged results are impacted less by the effects of outliers and high temporal variations in BC levels, with the Blue and IR BC readings for the MA350 and AE33 instruments producing r^2^ values of 0.810 and 0.888, respectively. For daily averaged values, the correlations between the MA350 and the AE33 increase with r^2^ values of 0.838 and 0.917 for Blue BC and IR BC, respectively. These findings at a mountainous, high-elevation, Greater Salt Lake City Area site support previous study results and broaden the body of evidence validating the performance of the MA350.

## Figures and Tables

**Table 1:** Key features of the AE33 [20] and MA350 [21].

	MA350	AE33
Wavelengths (nm)	375, 470, 528, 625, 880	370, 470, 520, 590, 660, 880, 950
Size	7 cm × 10 cm × 20 cm, 1 kg	28 cm × 43 cm × 33 cm, 21 kg
Flow	0.050–0.170 L min^−1^	2–5 L min^−1^
Detection Limit (IR BC)	0.030 μg m^−3^ (300 s, SingleSpot^TM^)	<0.005 μg m^−3^ (3600 s)
Timebase	1 s, 5 s, 10 s, 30 s, 60 s, 300 s	1 s, 60 s
Loading Effects Compensation	DualSpot^®^	DualSpot^®^
Battery	Yes	No
WiFi	Yes	No

**Table 2:** AE33 concerning status list and observed events. Note, a single measurement may have multiple status values.

Instrument Status	# Observed
Tape advance or fast calibration	2005
Stopped	1395
First measurement	1590
Flow out of range	0
Check status history	0
Calibrating led	5
Optical test	0
Optical calibration error	0
Led error	0
Tape error	1318
Stability test	4
Clean air test	68
Change tape procedure	2
Leakage test	66
Clean air test unacceptable result	0

**Table 3:** MA350 concerning instrument status list and observed events. Note, a single measurement may have multiple status values.

Instrument Status	# Observed
Tape advance	138
Start up	5
Flow unstable	213
Optical saturation	0
Sample timing error	0
Pump drive limit	0
User skipped tape advance	0
Tape jam	0
Tape at end	0
Tape transport not ready	0
Invalid date/time	0
Tape error	0

**Table 4:** Mean (St. Dev) loading-corrected values for the entire study period, 60 s timebase.

	MA350	AE33	Inter-Device Difference
IR BC (ng m^−3^)	534 (774)	474 (640)	11%
Blue BC (ng m^−3^)	651 (885)	591 (804)	9%
Biomass BC (ng m^−3^)	136 (227)	103 (183)	24%
Fossil fuel BC (ng m^−3^)	398 (519)	370 (530)	7%

## Data Availability

The data presented in this study are available on request from the corresponding author.
